# Biallelic variants in the *SORD* gene are one of the most common causes of hereditary neuropathy among Czech patients

**DOI:** 10.1038/s41598-021-86857-0

**Published:** 2021-04-19

**Authors:** P. Laššuthová, R. Mazanec, D. Staněk, L. Sedláčková, B. Plevová, J. Haberlová, P. Seeman

**Affiliations:** 1 Neurogenetic Laboratory, Department of Paediatric Neurology, Second Faculty of Medicine (2. LF UK) and University Hospital Motol, Prague, Czech Republic; 2 Department of Neurology, Second Faculty of Medicine (2. LF UK) and University Hospital Motol, Prague, Czech Republic; 3 Department of Paediatric Neurology, Second Faculty of Medicine (2. LF UK) and University Hospital Motol, Prague, Czech Republic

**Keywords:** Genetics, Neuroscience, Diseases, Molecular medicine, Neurology

## Abstract

Recently, biallelic variants in the *SORD* gene were identified as causal for axonal hereditary neuropathy (HN). We ascertained the spectrum and frequency of *SORD* variants among a large cohort of Czech patients with unknown cause of HN. Exome sequencing data were analysed for *SORD* (58 patients). The prevalent c.757del variant was tested with fragment analysis (931 patients). Sanger sequencing in additional 70 patients was done. PCR primers were designed to amplify the *SORD* gene with the exclusion of the pseudogene *SORD2P*. Sequence differences between gene and pseudogene were identified and frequencies of SNPs were calculated. Eighteen patients from 16 unrelated families with biallelic variants in the *SORD* gene were found and the c.757del was present in all patients on at least one allele. Three novel, probably pathogenic, variants were detected, always in a heterozygous state in combination with the c.757del on the second allele. Patients presented with a slowly progressive axonal HN. Almost all patients had moderate pes cavus deformity. SORD neuropathy is frequent in Czech patients and the third most common cause of autosomal recessive HN. The c.757del is highly prevalent. Specific amplification of the *SORD* gene with the exclusion of the pseudogene is essential for a precise molecular diagnostics.

## Introduction

Hereditary neuropathies (HN) are a group of disorders where neuropathy is the sole or primary cause of the symptoms^1^ and it is already known that pathogenic variants in more than 100 genes may cause HN^2^. Nevertheless, the cause of the disease remains unknown for many patients and mainly for patients with primary axonal neuropathy^3^.

Therefore, the recent remarkable observation of biallelic pathogenic variants in the *SORD* gene and a new possibly treatable form of axonal hereditary neuropathy (aHN) is very significant. Moreover, the authors proposed that the prevalent variant NM_003104.6(*SORD*):c.757del p.(Ala253Glnfs*27); is the commonest individual pathogenic allele in the biallelic state in hereditary neuropathies^4^.

In the past, homozygosity mapping in consanguineous families was used for the discovery of disease causing genes for autosomal recessive (AR) hereditary neuropathies^5,6^. Today, the utility of whole exome sequencing (WES) in the molecular diagnostics of AR rare diseases has been demonstrated^7^. However, there are still many challenges as illustrated in the SORD story. Recessive causal variants might have a relatively high population frequency in GnomAD^8^ or similar population databases, and their pathogenic character is therefore not easy to deduce. Actually, the population frequency of the variant might be the reason that the causal gene for a recessive disorder is overlooked for a long time.

At our Centre for neuromuscular disorders, our long-lasting interest is the elucidation of the causes of hereditary neuropathies and a detailed clinical characterization of many of the autosomal recessive forms of inherited neuropathies. Hence, the description of a new form of aHN with autosomal recessive inheritance and high frequency piqued our interest. Our cohort of patients is unique since from 1997 patients from the whole of the Czech Republic have been tested in our DNA laboratory. For about 20 years, we were the sole diagnostic laboratory for HN in our country; and because of our unique position, we have been able to collect more than 3000 DNA samples from patients with HN (or probable HN), from the whole Czech Republic. To date, we have clarified the cause of HN at the DNA level in 2313 patients. In addition, our population is specific as we have one of the largest cohorts of patients with specific autosomal recessive subtypes of inherited peripheral neuropathies, for example, with disease causing variants in *HINT1*, *SH3TC2*, *FIG4*, *HK1*, *NDRG1* and others^9–13^.

In this study, we performed extensive screening for the presence of *SORD* pathogenic variants in Czech patients with unclarified, probably hereditary peripheral neuropathies. We established the spectrum and frequencies of variants in the *SORD* gene in the Czech population and the relative contribution of the *SORD* gene to the diagnostics of HN in the Czech Republic.

## Patients and methods

All patients signed an informed consent form and the ethical committee of the Motol University Hospital approved the study. All methods were carried out in accordance with relevant guidelines and regulations.

### Criteria for patients selection

In total four different analyses were done. Firstly, in 58 unrelated patients with an unknown cause of HN and in whom we already had previous whole-exome sequencing (WES) data, we re-analyzed WES data for variants in the *SORD* gene. Secondly, we used screening with fragment analysis to search for the c.757del variant in a large cohort of 931 patients. Basically, all patients from our database, without clearly autosomal dominant inheritance and without a known cause of their disease were selected. From the 931 selected patients, 41% (387) have axonal neuropathy, 16% (152) demyelinating and 6% (52) intermediate type of HN. The information was not available for 18% (165) of the patients and the type of neuropathy was undecidable based on the available documentation in 19% (175) of patients (details are presented in the Supplementary file 1). These patients had an unknown cause of the disease, and most of the patients had been tested previously for CMT1A/HNPP or other more frequent causes of hereditary neuropathies depending on their phenotype or provided clinical and electrophysiological information. All *SORD* variants found by WES or fragment analysis were confirmed by Sanger sequencing in the patient and also in available family members. Finally, we continued with Sanger sequencing for additional, carefully selected patients - based on their phenotype; we searched for clinical signs described in^4^ - axonal type of HN (HMSN II or HMN) and compatible with recessive inheritance. In total, we sequenced exon 7 in 70 patients. From these, a subgroup of 32 patient was selected for sequencing of all remaining coding exons of the *SORD* gene. With this workflow, we searched for variants other than the c.757del, which is located in the exon 7.

**Figure 1:**
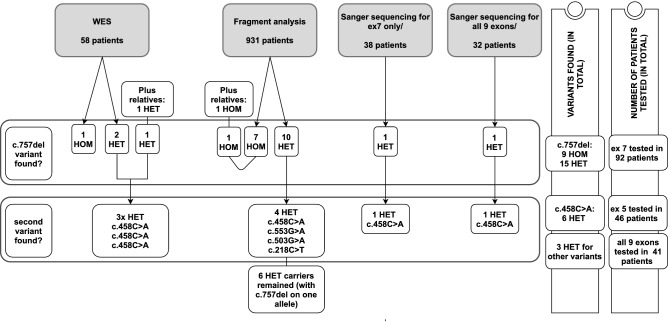
Workflow of the study. Four different analyses were done. In total, biallelic *SORD* variants were identified in 18 patients from 16 unrelated families. Prevalent variant is the c.757del.

The outline of the workflow of the study is summarized in Fig. 1. In total, four different analyses were done.

1. In 58 unrelated patients with an unknown cause of HN, in whom we already had whole-exome sequencing (WES) data from the past, we re-analyzed WES data for variants in the *SORD* gene. If a variant was observed, it was confirmed by Sanger sequencing (three patients).
2. In 931 unrelated patients with an unknown cause of HN (regardless of the type of neuropathy, but compatible with the recessive mode of inheritance, and previously tested for relevant causes of HN) screening for the presence of the c.757del variant by fragment analysis of a fluorescent PCR product was performed. A part of exon 7 was PCR-amplified with a fluorescently labelled primer specific for the *SORD* gene, then the fragments were separated by capillary electrophoresis and analyzed on an ABI 3130 Genetic Analyzer (ThermoFischer Scientific). The workflow, including primer sequences and the methodology, is illustrated in the Supplementary file 1. All seven c.757del homozygotes were subsequently confirmed by Sanger sequencing of exon 7 of *SORD*. In all of the 10 detected c.757del heterozygotes, further Sanger sequencing was done in order to identify the second causal variant. Moreover, similarly affected siblings (2 in two families) were included in the testing.
3. In additional 32 unrelated patients, Sanger sequencing of all nine coding exons of the *SORD* gene was performed with the use of gene-specific primers. These patients were carefully selected from our database of patients based on their phenotype according to the published data; we searched for clinical signs described in^4^: axonal type of HN (HMSN II or HMN) and compatible with recessive inheritance.
4. In additional 38 patients, Sanger sequencing of exon 7 of *SORD*, with the prevalent variant c.757del, was performed. This group of patients was also selected from our database according to the published clinical signs^4^.

In total, WES data were re-analyzed for *SORD* in 58 patients and a fragment analysis was performed in 931 patients. Exon 7 of the *SORD* gene was sequenced in 92 patients in total (3 from WES; 17 from fragment analysis; 2 from additional family members in two families with similarly affected siblings; 32 patients selected from our database for Sanger sequencing of the entire coding region; and 38 different patients selected only for Sanger sequencing of exon 7 of the *SORD* gene). Then, exon 5 of the *SORD* gene was sequenced in 46 patients in total (12 heterozygotes for c.757del from WES and a fragment analysis of 1 affected sibling and 1 heterozygous carrier of c.757del from the 38 patients selected for exon 7 sequencing). In patients where the pathogenic variant on the second allele was found (c.458C>A p.(Ala153Asp); number of patients = 6) these patients were not tested further. However, for the remaining 41 patients, all 9 exons of the *SORD* gene were Sanger sequenced. This is summarized in Fig. 1.

For PCR amplification and Sanger sequencing, care was taken to amplify specifically the *SORD* gene and not the pseudogene *SORD2P*. To achieve this, primers for PCR amplification were designed in such a way that the primer sequence was located in a region with differences between the *SORD* and *SORD2P* sequences. In addition, it was important that primers were not located at the site of frequent polymorphism. As per the original publication^4^, the primers for exons 1–6 and 9 fulfilled these criteria, however, for exons 7 and 8 we recommend different primers are used.

For exon 7 of the *SORD* gene, the internal sequencing primer (5′-AAAAGAAAACATAGATGGCAAAAGA-3′) from the original publication is located at the site of frequent SNP: Chr15(GRCh38):g.45069181A>C and with a frequency of 21.94% in gnomAD (genomes), may cause an allele drop out that leads to a false result.

Therefore, we used a new internal sequencing primer with the sequence 5′-GCTCACGCAGCAAGCTGGTAAAG-3′ instead.

**Figure 2:**
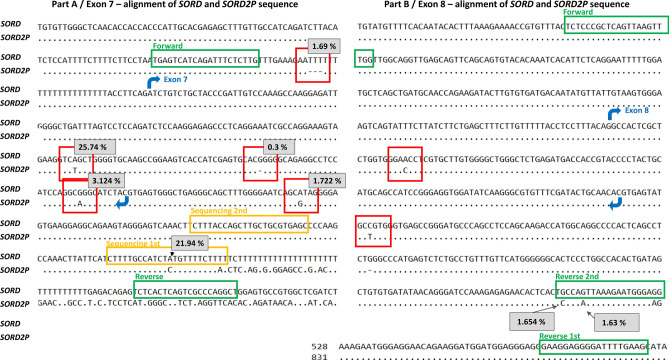
The sequence differences of *SORD* and *SORD2P*. Blast search for *SORD* and *SORD2P* with alignment view pairwise with dots for identities (https://blast.ncbi.nlm.nih.gov). Several features are highlighted: red squares = differences; green rectangles = PCR primers; orange rectangles = sequencing primer; blue arrow = start and end of the exon; 1st primer = primer from original study; 2nd primer = our primer; grey rectangles with % = population frequency of that SNP in gnomAD for *SORD.*

In addition, for a reliable discrimination between the gene and the pseudogene, a combination of three SNPs should be used. This is illustrated in Fig. 2. There are five differences in the sequence between *SORD* and *SORD2P* in the amplified region of exon 7. For reverse control of gene amplification, we recommend using a combination of all three SNPs, which are present in the *SORD* but not the *SORD2P* sequence.

To validate the five sequence differences in the *SORD* and *SORD2P* exon 7 sequence, we calculated the frequency of these SNPs in our patients by Sanger sequencing of exon 7.

Moreover, in the original publication, the PCR primers used for the amplification of exon 8 were not specific for *SORD*, as they also amplified the *SORD2P* exon 8. Therefore, we designed a different reverse primer to achieve a specific amplification with the sequence 5′-TCCTCCCATTCTTTAACTGGC-3′. This is illustrated in Fig. 2B.

The amplification and thermocycling protocols were used from the original paper. As for the chemistry, we used a CombiPP mix from TopBio (http://www.top-bio.cz/). For details, see the Supplementary methods section.

### Research methods

All methods were carried out in accordance with relevant guidelines and regulations.

### Ethics declaration

Research was conducted according to the principles expressed in Declaration of Helsinki and was approved by the local Ethics committee (University Hospital Motol). All participants or in case of children their legal representatives provided informed consent for the use of clinical data, collection of samples and subsequent DNA analyses. All patient’s data are presented in the manuscript in an anonymized form.

## Results

### Ballelic pathogenic, or likely pathogenic variants, in the *SORD* gene were detected in 18 patients from 16 unrelated families

Nine patients were homozygous for the c.757del variant  p.(Ala253Glnfs*27); nine patients were compound heterozygous for c.757del and a different variant on the second allele of the *SORD* gene (6 × c.458C>A p.(Ala153Asp), 1 × c.218C>T p.(Ser73Leu), 1 × c.503G>A p.(Gly168Asp), 1 × c.553G>A p.(Gly185Arg)). There were six heterozygous carriers of the c.757del variant in whom no second variant was detected in the *SORD* gene despite Sanger sequencing of all nine *SORD* exons. This is illustrated in Fig. 1.

Firstly, WES data from 58 patients were reanalysed for variants in the *SORD* gene. Biallelic pathogenic variants were detected in three patients (5.2%) - one homozygote for c.757del and two compound heterozygotes for c.757del and c.458C>A.

Secondly, a DNA fragment analysis for the presence of c.757del was performed in 931 patients. The c.757del variant was detected on at least one allele in 17 patients (1.8% for the whole group = 931, this corresponds to 4.4% among patients with axonal neuropathy = 17/387)- seven were homozygous, and 10 were heterozygous. Confirmation of c.757del with Sanger sequencing of exon 7 of the *SORD* gene was done for all of them.

In addition, Sanger sequencing of exon 7 was done in an additional 70 selected (32 + 38) patients from our database. Additional two c.757del heterozygotes were detected (2.8%).

At this point, 9 (incl. one sibling) homozygotes and 15 heterozygotes (incl. one sibling) for c.757del were detected by the above-mentioned tests. All 15 heterozygotes for the c.757del were further tested by exon 5 Sanger sequencing. As previously described, c.458C>A was detected in a total of six patients, always in a heterozygous state and in combination with a c.757del on the second allele. In the remaining 9 heterozygotes (15 minus 6), Sanger sequencing of all remaining exons of the *SORD* was done and the second probable causal variant on the second allele of the *SORD* gene was found in three of them (1 × c.218C>T p.(Ser73Leu), 1 × c.503G>A p.(Gly168Asp), 1 × c.553G>A p.(Gly185Arg)).

In an additional 32 selected patients, all nine coding exons of *SORD* were sequenced and no additional variants were detected.

### The spectrum and frequency of detected *SORD* variants

The most frequent variant (79% of pathogenic alleles) was the previously described c.757del detected in 33 alleles. The second most frequently detected (14% of pathogenic alleles) was the c.458C>A (also previously described as pathogenic) in 6 alleles. Furthermore, three novel missense variants were detected - always on one allele only (2%) in combination with the pathogenic and prevalent c.757del variant on the second allele. All 18 patients with biallelic, pathogenic or probably pathogenic variants in *SORD* carry the c.757del on at least one allele (either homozygous or heterozygous state - in combination with a second pathogenic or probably pathogenic variant in *SORD*). Therefore testing for the prevalent variant with subsequent sequencing of all nine coding exons in heterozygotes only should be able to detect all or nearly all patients with biallelic pathogenic variants in *SORD*.

**Figure 3:**
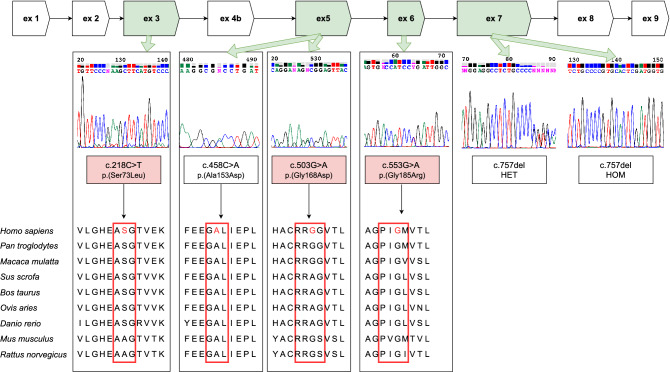
Variants in the *SORD* gene detected in this study. Upper part - The structure of the *SORD* gene with nine coding exons. Middle part - Sanger sequences of variants detected in the study; detected novel variants are in pink. HET - hetrozygous; HOM-hoomzygous. Lower part - The conservation of amino acids based on a multiple sequence alignment analysis for missense variants detected in the study (https://www.ebi.ac.uk/Tools/msa/).

**Figure 4:**
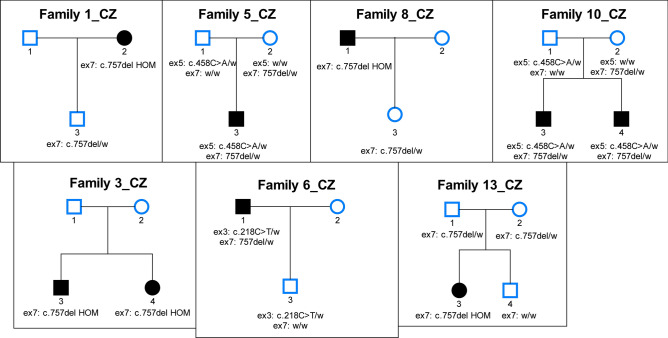
The pedigrees of selected families. *HOM* homozygous, *w* wild type; Pedigrees for families where at least two family members were tested are shown.

The characterization of the novel variants in exons 3, 5, and 6 is summarized in Table 1. The localization of variants in the *SORD* gene, electropherograms and an evaluation of conservation among the different species of these variants are presented in Fig. 3. The pedigrees of the selected families are shown in Fig. 4. In summary, nine patients were compound heterozygous for the c.757del variant and a missense variant. A segregation analysis was possible in five of them. In families 5 and 10 the parents were tested. In family 6 one offspring was tested. A segregation analysis was not possible for patients from families 7, 9, 11, 15 and 16; patients from families 7, 15 and 16 are compound heterozygous for c.[458C>A];[757del] - both variants were previously described as causal. However, a patient from family 9 is heterozygous for c.[553G>A];[757del] and a patient from family 11 is heterozygous for c.[503G>A];[757del]. A segregation analysis was not possible for these two families.

### The contribution of *SORD* variants among other known causes of HN in Czech patients with a clarified cause of HN

**Table 1:** The characterization of novel *SORD* variants detected in this study (Alamut Visual version 2.15 (SOPHiA GENETICS, Lausanne, Switzerland)).

cDNA Level (NM_003104.6): gDNA Level Chr15 (GRCh38): Protein Level:	c.218C>T g.45043374C>T p.(Ser73Leu)	c.503G>A g.45065348G>A p.(Gly168Asp)	c.553G>A g.45068189G>A p.(Gly185Arg)
Nucleotide conservation (phyloP: [-14.1;6.4])	Highly conserved (phyloP: 5.21)	Weakly conserved (phyloP: 0.93)	Highly conserved (phyloP: 5.05)
Amino acid conservation	Moderately	Weakly	Highly
Physiochemical difference (GD [0–215])	Large (GD: 145)	Moderate (GD: 94)	Moderate (GD: 125)
SIFT (v6.2.0)	Deleterious	Deleterious	Deleterious
MutationTaster (v2013)	Disease-causing (prob: 1)	Disease-causing (prob: 0.967)	Disease-causing (prob: 1)
dbSNP (151)	rs765897771	rs760749907	rs753424622
gnomAD (2.1)	ALL:0.0031% - AFR:0.026%	ALL:0.00040% - NFE:0.00090%	ALL:0.0039% - AFR:0.0040% - SAS:0.0033% - NFE:0.0070%

*GD* Grantham distance; *prob* probability.

Our results, from a large collection of Czech patients with unclarified cause of presumable hereditary neuropathy, confirm that biallelic pathogenic variants in the *SORD* gene form a significant proportion of autosomal recessive causes of HN. Furthermore, although among Czech patients it is not the most common autosomal recessive form of HN, it is the third most frequent cause of HN (after *HINT1* and the *SH3TC2* variants with 27 and 24 families, respectively). The phenotype of patients with *SORD* bialellic variants is recognisable and we recommend screening for the prevalent c.757del variant in all unclarified HN patients, since it is an easy test to perform.

The relative contribution HN caused by biallelic variants in *SORD* to the whole spectrum of hereditary neuropathies with a known molecular cause among Czech patients was calculated. Since our database was established in 1997, we have clarified 2313 patients from 1171 families that have a known cause of hereditary neuropathy - with causal pathogenic variants (on average about 100 clarified patients per year). The eighteen patients we detected with biallelic *SORD* variants account for 0.78% (18 patients out of 2313) of all diagnosed patients and 1.36% of all families for which the cause of their neuropathy had been stated (16 families from 1171).

### The clinical features of patients with the SORD neuropathy

The clinical features are summarized in the tables below.

In our patients with biallelic pathogenic variants in the *SORD* gene, the median age of onset was 15 years (SD ± 12.57y). The median age at examination was 55 years (SD ± 15.35y). There were 11 males and seven females; most of the patients were sporadic cases (12 families, 75%) and in four families, there were similarly affected siblings (Table 2).

**Table 2:** The characterization of patients with SORD neuropathy.

Family	Individual#	Genotype		Age		Sex	Family history	Type of neuropathy	EMG		Age at EMG examination (years)
		Allele 1	Allele 2	At onset (years)	At molecular diagnosis (years)				MNCV (m/s)	SNCV (m/s)	
1	2	c.757del	c.757del	15	51	F	Sporadic	HMSN II	42.0	50	30
2	1	c.757del	c.757del	NA	59	M	Sporadic	HMSN II	52.9	NA	41
3	3	c.757del	c.757del	0–10	67	M	Familiar – sister	HMSN II	NA	NA	NA
	4	c.757del	c.757del	30	70	F	Familiar – brother	HMSN II	46	50	54
4	1	c.757del	c.757del	40	59	M	Sporadic	HMSN II	NA	NA	42*
5	3	c.757del	c.458C>A	13	30	M	Sporadic	HMSN II	NA	NA	NA
6	1	c.757del	c.218C>T	10–20	75	M	Familiar – sibling (DNA NA)	HMSN (INTER)	50	NA	49
7	1	c.757del	c.458C>A	20–30	61	F	Familiar – two siblings (DNA NA)	HMSN II	56.1	63.8	47
8	1	c.757del	c.757del	10	59	M	Sporadic	dHMN	53.2	48.5	55
9	1	c.757del	c.553G>A	49	53	M	Sporadic	HMSN II	NA	NA	46
10	3	c.757del	c.458C>A	0–5	23	M	Familiar – brother	HMSN II	52.9	NA	13
	4	c.757del	c.458C>A	5–10	21	M	Familiar – brother	HMSN II	27.1	41.7	15
11	1	c.757del	c.503G>A	20–25	60	M	Sporadic	HMSN II	NA	NA	42*
12	1	c.757del	c.757del	13	22	M	Sporadic	HMSN (INTER)	55.2	50	15
13	3	c.757del	c.757del	0–5	30	F	Sporadic	HMSN II	NA	NA	25*
14	1	c.757del	c.757del	13	37	F	Sporadic	HMSN II	36.0	42	31
15	1	c.757del	c.458C>A	10–20	38	F	Sporadic	dHMN	NA	NA	38*
16	1	c.757del	c.458C>A	51	57	F	Sporadic	HMSN II	NA	NA	56

^#^Individual, this number corresponds with the number in the Fig. 4 (Pedigrees); where no additional family members were available # = 1; *F* female; *M* male; *NA* not available; *HMSN II* axonal type of neuropathy; *INTER* Intermediate type of HN; *dHMN* distal hereditary motor neuropathy; *EMG* electromyography; Nerve conduction velocities for n. medianus:* MNCV* motor nerve conduction velocity; *SNCV* sensory nerve conduction velocity. *EMG exam was done, but results are available only in the form of a commentary (not numerical values).

Clinically, we observed slowly progressive distal peripheral neuropathy, primarily axonal, on all extremities (Table 3). All patients presented with axonal neuropathy, motor and sensory (HMSN II) or initially, and in younger patients, as pure motor axonal neuropathy (dHMN) or as an intermediate form of HMSN. Pes cavus was present in almost all adult patients (14/15). For one patient no signs of foot deformity were observed at the age of 38 years. Moreover, distal muscle weakness and atrophy were the main neuropathic symptoms, with atrophy of the calves present in all patients examined. For some patients, hypotrophy of the thighs was also prominent (4/11). Where patient information was available, the reflexes L2-L4 were decreased, and the L5-S2 were absent in most of the patients.

**Table 3:** A summary of the clinical features of patients with biallelic pathogenic variants in the *SORD* gene.

Clinical feature	Number of patients with clinical features/total number of tested individuals
**Pes cavus deformity**	14/15
**Lower limb atrophy**	11/11
**Lower limb weakness**	
Proximal	5/8
Distal	15/15
**Lower limb reflexes (L2-L4)**	
Decreased	8/12
Absent	3/12
**Lower limb reflexes (L5-S2)**	
Decreased	1/13
Absent	11/13
**Use of walking aids**	1/10
**Upper limb atrophy**	7/14
**Upper limb weakness**	
Proximal	1/8
Distal	8/13
**Upper limb reflexes**	
Decreased	7/10
Absent	1/10
Light tremor of hands*	3
**Sensory impairment**	
Loss of vibration	10/13
Tactile hypesthesia	4/13
Thermic hypesthesia	4/13
Impairment of recognition of sharp and dull objects	1/13
Paresthesia	1/13
Scoliosis*	1

**Total individuals tested = 18.**

In total, 18 patients were included. The number of patients with a particular clinical sign are shown in the second column. The total number of patients tested for a particular sign is shown after the forward slash. For some of the patients, the information was not available. For some clinical signs - marked with *- only the number of patients with positive signs is given.

## Discussion

### The differentiation of the *SORD* and *SORD2P* sequences

The *SORD* gene has a pseudogene, *SORD2P,* with high homology (the identity is 2295 out of 2320 bases with three gaps). Pseudogenes are usually not actively transcribed or translated, and they can be recognised by the presence of a stop codon or a frameshift that interrupts the open reading frame^14,15^. This is true for *SORD2P* where the c.757del variant is normally present, and thus results in a premature stop codon and a non-functional protein. In addition, this supports the idea that the c.757del variant in the *SORD* gene results in a non-functional protein, as well.

However, in molecular genetic testing and diagnostics, reliable elimination of *SORD2P* sequences is very challenging.

In order to avoid amplification of the pseudogene, and all that entails, we adopted and validated a specific primers for PCR and Sanger sequencing. It is well known that a variation in the last three bases at the 3′ end of the primer sequence can particularly disrupt primer hybridisation and decrease PCR efficiency^16^. This feature can be used to obtain specific primers for genes with homologous pseudogene regions/genes. Specific primers should be chosen with mismatches at the 3′ end^17^. On the other hand, because the presence of frequent SNPs at the primer site may cause allelic dropout, primers need to be designed specifically to avoid frequent SNPs (population frequency > 1%) at the primer site.

Based on this, we recommend that the internal sequencing primer for exon 7 published in the original paper^4^ should not be used, because there is a frequent SNP at the primer site, which may cause allele drop out and lead to incorrect results. Furthermore, published primers for exon 8 of the *SORD* gene also co-amplify the pseudogene, *SORD2P.* For exon 7, this is illustrated in Fig. 2.

Another point from the original paper should be mentioned: to discriminate between *SORD* and *SORD2P,* the authors used these two SNPs in exon 7: Chr15(GRCh38):g.45068982A>T and Chr15(GRCh38):g.45069043G>A. However, in the *SORD* gene in the GnomAD genome database, both SNPs have a high frequency (25.7% and 1.77% respectively). Therefore, heterozygotes and even a few homozygotes may occur. And indeed, we have seen repeatedly that our samples carried those SNPs in the *SORD* exon 7, and our observed frequency for Chr15(GRCh38):g.45068982A>T was 17.45%. This is illustrated in Table 4.

**Table 4:** Frequencies of SNPs in exon 7 of the *SORD* gene in our samples.

Variant	Number of observed homozygotes	Number of observed heterozygotes	Number of alleles	% of alleles
NM_003104.6:c.777G>A p.(Ala259Ala) Chr15(GRCh38):g.45069043G>A	1	13	15	7.08
NM_003104.6:c.716A>T p.(Gln239Leu) Chr15(GRCh38):g.45068982A>T	10	17	37	17.45
NM_003104.6:c.757del Chr15(GRCh38):g.45069023del	9	16	38	17.92
Total number of patients tested 106			Total number of alleles tested 212	

Based on our observation, we recommend the use of different SNPs for the discrimination of gene vs pseudogene, as using these two SNPs with a high population frequency might be confusing. Our proposed workflow is as described in the method section and illustrated in Fig. 2.

Accordingly, we recommend using different sites for reliable elimination of pseudogene sequences, or a combination of all differences that are in the amplified region. In total, there are five differences and one of them is the c.757del variant. The combination of these three SNPs might be very effective for the control of specific *SORD* amplification: Chr15(GRCh38):g.45069043G>A, Chr15(GRCh38):g.45069087A>G, and Chr15(GRCh38):g.45068867_45068868del (illustrated in Fig. 2) – but not Chr15(GRCh38):g.45068982A>T.

### NGS data analysis

For NGS data analysis, two steps should be considered for distinguishing between the *SORD* gene and the *SORD2P* pseudogene. These are: define the region of interest (target) properly (proper selection of the target region is crucial) and optimize the parameters in the analysis pipeline. However, amplification and hybridisation are done most often with commercially available kits, so it is not possible to optimize them in such a way as to improve the discrimination between genes and a pseudogene. In addition, sequencing with short reads (for ex. Illumina) is prone to ambiguous mapping, but this might be improved with pair-end sequencing which is highly recommended. The analysis pipeline has to be optimized for gene/pseudogene discrimination. In our study we applied the workflow summarized by Claes, et al.^17^, and three points should be highlighted here: seed length; gap penalty; and base quality score. Our NGS analysis was done according to the GATK Best practices recommendations^18,19^. For alignment, BWA mem was used, with seed length and gap penalty set to the default values. More information is available here: http://bio-bwa.sourceforge.net/bwa.shtml. Shorter reads are deleted and reads with gaps longer than expected are excluded. Furthermore, for base quality recalibration, we used BQSR (https://gatk.broadinstitute.org/hc/en-us/articles/360035890531-Base-Quality-Score-Recalibration) according to the GATK best practices. In every case, variants detected with NGS have to be confirmed by Sanger sequencing, and with specific PCR primers, in order to avoid coamplification of pseudogene sequences.

Our study of 18 newly detected patients with biallelic pathogenic, or probably pathogenic disease causing variants, in *SORD*, all with the c.757del disease causing variant on at least one allele, confirms also in a large sample of Czech patients the importance of the recent discovery of this new disease-causing gene and its prevalent disease causing variant for hereditary neuropathy^4^.

The recognition of the biallelic *SORD* variants as causal for axonal hereditary neuropathy allows for a significant increase in the diagnostic rate of aHN, especially the autosomal recessive and axonal types.

### Total number of patients with SORD neuropathy and variants detected

On the other hand, as we were able to test a large number of patients from the same population and same collection of patients of a similar size, we have observed that biallelic disease causing variants in *HINT1* or *SH3TC2* are still a more frequent cause of HN than *SORD*. We have found 16 families with biallelic disease causing variants in *SORD*, but 27 families with *HINT1* bialellic disease causing variants and 24 with *SH3TC2* bialellic disease causing variants. For all three genes, we performed a very similar extensive screening of about 800 to 900 patients from our registry, collected since 1997, that had a neuropathy without a known cause. Therefore, we conclude that SORD related neuropathy is one of the most important, or the third most common, type of autosomal recessive HN, at least in the Czech population.

Clinically, the patients presented with axonal neuropathy and, in almost all of them, mild to moderate pes cavus deformity was present. Most often, the first signs of the disease occurred in their second decade of life and the disease was slowly progressive. The muscle atrophy and weakness of peroneal and calf muscles, mild to moderate foot deformities (pes cavus) are obvious. The sensory impairment of the lower limbs is relatively mild compared to the motor deficit, but the majority of patients are still able to walk unaided in the advanced stages of the disease.

To search for all possible *SORD* variants, and to establish the real spectrum of variants in the Czech population, we sequenced all nine coding exons of *SORD* in 99 Czech patients; 41 by Sanger sequencing and 58 reanalyzed using their WES data. However, no patients with biallelic pathogenic *SORD* variants without the c.757del in at least one copy were detected. Three novel probable pathogenic variants were detected in this study, always in combination with a prevalent c.757del variant on the second allele.

The prevalent pathogenic disease causing variant c.757del was present in all 18 patients with a biallelic pathogenic variant on at least one allele (heterozygous or homozygous state).The same was described in the original study with 45 individuals. Therefore, by screening for the c.757del only we should be able to detect all or nearly all patients with a SORD neuropathy.

In our study, four different analyses were done. WES data from 58 patients were reanalysed and three patients turned out to carry biallelic pathogenic *SORD* variants (5.2%). However, this is a very carefully preselected group of patients, as WES is done only after thorough full evaluation. Results from the fragment analysis with 931 patients tested represent, in our opinion, much better the true distribution of *SORD* variants in Czech patients with neuropathy. From a group of all unclarified patients without clear autosomal dominant inheritance, 17 carry the c.757del on one allele at least (1.8%). In addition, from these 17 patients, six were heterozygous carriers without second causal variant found (0.6%) and 11 patients have biallelic causal SORD variants (1.2%). If further larger scale studies are done, it might be interesting to see, if this is true also for other populations.

### Other published cases of SORD neuropathy

Recently, biallelic *SORD* variants were described in four novel studies. Yuan, et al. described three patients, two homozygous for the c.757del variant and one compound heterozygous for the c.757del and c. 625C>T variant which lead to p.(Arg209*)^20^. Clinically, patients presented with axonal neuropathy, lower limb weakness and atrophy. The first signs of the disease occurred at puberty (14–16 years). The study by Dong et al. presented four Chinese patients^21^. Three of them are homozygous for the c.757del variant and one is compound heterozygous for c.404A>G p.(His134Arg) and c.908 + 1G>C, which is a splicing variant. In addition, the authors showed, that the variant results in the abolition of the donor splice site. The phenotype is very similar to other published cases with onset in the second life decade. Lower limb weakness and distal muscle atrophy are the main presentation. This paper is interesting, because it is the first published case of a patient who is compound heterozygous for c.404A>G and c.908 + 1G>C without the c.757del variant. Thirdly, a comprehensive paper by Frasqet, et al. describes a mutational spectrum in a large cohort of 163 patients with distal motor hereditary neuropathy^22^. Five patients with biallelic *SORD* variants are presented; one is homozygous for the c.757del variant, and the remaining four patients, from two unrelated families, are compound heterozygous for c.757del and c.458C>A. Two sporadic cases homozygous for c.757del are described^23^. Patients presented with childhood disease onset and mild phenotype. These cases further support that c.757del is the prevalent pathogenic variant. Authors concluded, that further - large scale population study - is needed to establish the proper spectrum of SORD related neuropathy in China.

The phenotype of all the patients presented in abovementioned papers in quite homogenous. The onset of the disease was in the second/third life decade. Patients present with axonal neuropathy, distal muscle weakness and atrophies. All are homozygous or compound heterozygous for the c.757del except for one patient.

**Figure 5:**
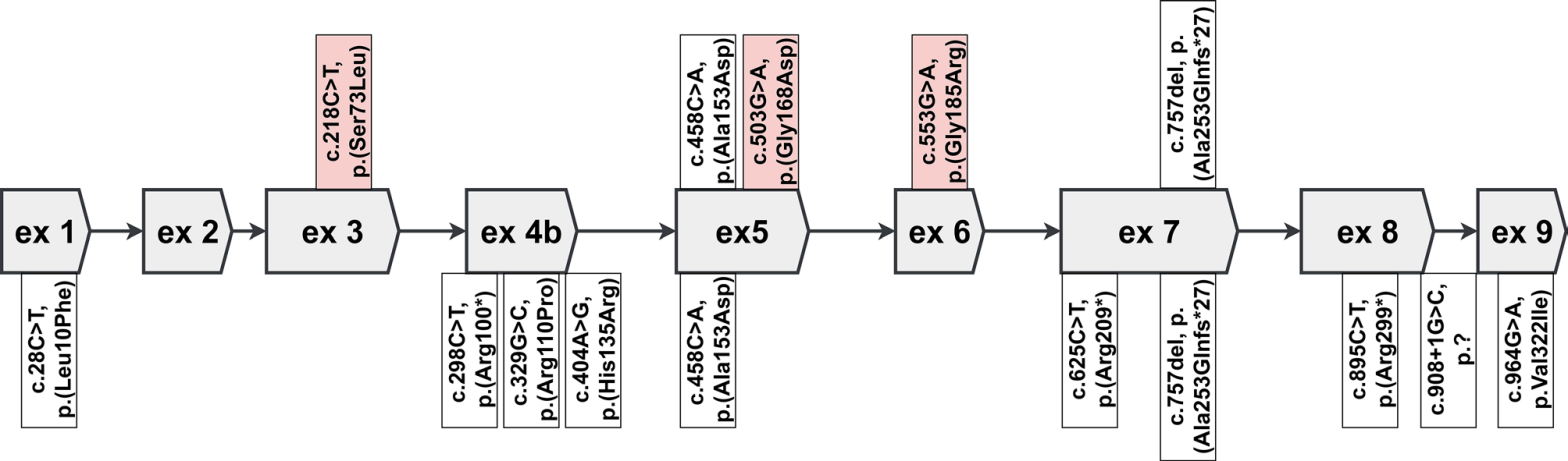
The *SORD* gene structure and published variants. Above the gene: variants found in this study. Below the gene are previously published variants. Red flags describe novel variants found in this study.

All variants, detected up to date, are summarized in Fig. 5.

## Summary

Our results show that biallelic pathogenic variants in the *SORD* gene are a very important cause of HN in Czech patients and are one of the most common causes of autosomal recessive HN. The c.757del disease causing variant is also highly prevalent in the Czech population and this can be used for very effective diagnostics. Our study confirms the significance of biallelic pathogenic variants in the *SORD* gene for hereditary neuropathies worldwide. Because of the high prevalence of the c.757del disease causing variant, we recommend screening of this variant in all patients without clear dominant family transmission, regardless the type of neuropathy. For Sanger sequencing, special care should be taken to reliably and specifically amplify only the *SORD* gene and elimiante the pseudogene *SORD2P*. For NGS data analysis, proper analysis parameters must be tweaked, especially in regard to alignment settings.

### Key findings

1. It is important to discriminate precisely between the *SORD* and *SORD2P* sequences. We present a suitable workflow and discuss the possible pitfalls of SNPs used for discrimination. Such a comparison has not been published or discussed, up till now.
2. Biallelic variants in the *SORD* gene are a frequent cause of hereditary neuropathy. SORD neuropathy is one of the most important types of autosomal recessive HN. We did extensive screening and tested the largest cohort for the presence of the prevalent c.757del variant. Nobody so far has tested such a large cohort of patients with HN for the presence of the c.757del variant.
3. The phenotype is quite uniform: Axonal neuropathy, with muscle weakness and distal lower limb atrophy is present in almost all patients. The onset of the disease is usually in the second or third life decade. This is concordant with other published studies.

## Supplementary Information

Supplementary Information.

## Data Availability

All protocols are available upon request. Online resources: https://blast.ncbi.nlm.nih.gov, https://www.ebi.ac.uk/Tools/msa/.
