# Supplementary material for: Biallelic variants in the SORD gene are one of the most common causes of hereditary neuropathy among Czech patients
Source: Sci Rep. 2021 Apr 19;11:8443. doi: 10.1038/s41598-021-86857-0 (PMC8055917; doi:10.1038/s41598-021-86857-0)
Supplement: Supplementary file 1 — Supplementary Information. [file 41598_2021_86857_MOESM1_ESM.pdf]

Supplementary file for manuscript:

Biallelic variants in the *SORD* gene are one of the most common causes of hereditary neuropathy among Czech patients

Author list:

P.Laššuthová[1*], R.Mazanec[2] , D.Staněk[1], L.Sedláčková[1], B.Plevová[1,3], J. Haberlová[3], and P.Seeman[1]

Affiliations:

1- Neurogenetic laboratory, Department of Paediatric Neurology, Second Faculty of Medicine (2. LF UK) and University Hospital Motol, Prague, the Czech Republic

2- Department of Neurology, Second Faculty of Medicine (2. LF UK) and University Hospital Motol, Prague, the Czech Republic

3- Department of Paediatric Neurology, Second Faculty of Medicine (2. LF UK) and University Hospital Motol, Prague, the Czech Republic

* corresponding author

Contact information for corresponding author

Petra Laššuthová, M.D., Ph.D.
Neurogenetic lab
Department of Paediatric Neurology
Second Faculty of Medicine, Charles University in Prague and
Motol University Hospital

Contact details:
V Úvalu 84, 150 06 - Prague 5
The Czech Republic

Phone: +420224436788

Email: petra.lassuthova@fnmotol.cz

Content

Biallelic variants in the *SORD* gene are one of the most common causes of hereditary neuropathy among Czech patients 1
1. Supplementary methods 3
1.1. Patients 3
1.1.1. Patient selection criteria 3
1.2. Methods 4
1.2.1. DNA fragment analysis by capillary electrophoresis 4
1.2.2. Sanger sequencing of the *SORD* gene 6
1.2.3. Analysis of WES data 10

Figures:

Figure 1: Characterization of types of neuropathy for patients selected for fragmentation analysis.... 3
Figure 2**:** Fragment analysis results 5
Figure 3**: PCR products for exon 8 of the SORD/SORD2P** 7
Figure 4**: Electropherograms of SORD exon 7** 9

Tables:

Table 1: Type of neuropathy among patients tested with fragment analysis 3
Table 2: Primers used for fragment analysis 4
Table 3: Primers used in this study 6
Table 4: Other variants detected with WES in patients with biallelic SORD disease causing variants. 11

1. Supplementary methods

1.1. Patients

1.1.1. Patient selection criteria

Fragment analysis was used as a screening method for the prevalent c.757del variant. Therefore, we opted for the testing of as many patients with inherited peripheral neuropathy, as possible. In fact, we tested all the available DNA samples without autosomal dominant inheritance in the family and with an unclarified cause of the disease from our database.

From the 931 selected patients, 41% have axonal neuropathy, 16% demyelinating and 6% intermediate. The information was not available for 18% of the patients and the type of neuropathy was undecidable based on the available documentation in 19% of patients. This is shown in table 1 and in figure 1, below.

Type of neuropathy	Number of patients
Axonal	387
Demyelinating	152
Intermediate	52
Undecidable	175
N.A.	165
**Total selected**	**931**

Table 1: Type of neuropathy among patients tested with fragment analysis

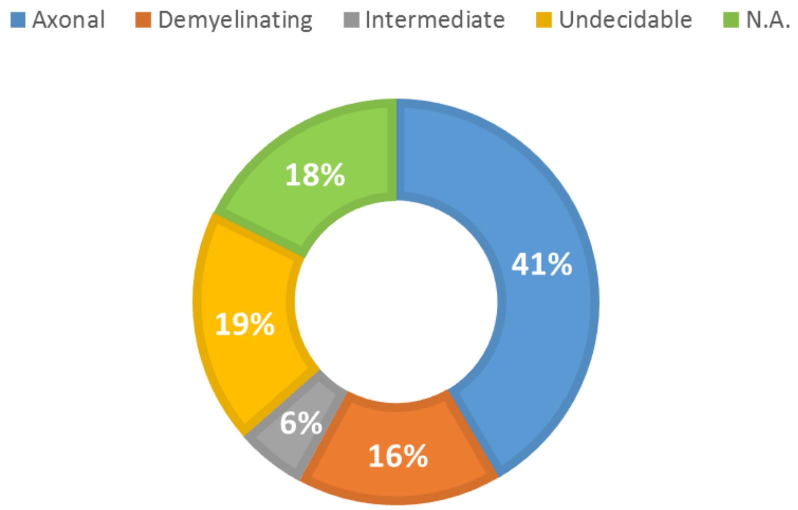

Figure 1: Characterization of types of neuropathy for patients selected for fragmentation analysis

Legend: Undecidable – not possible to decide based on available information from electromyography study; N.A. – no information available from the nerve conduction study

1.2. Methods

1.2.1. DNA fragment analysis by capillary electrophoresis

The principle of fragment analysis is amplification of the target DNA with labelled primers. Gained products are then separated by capillary electrophoresis and the relative size of each product is determined. Accordingly, it is possible to state a genotype of a sample.

For our fragment analysis these primers were used:

Forward primer labelled with FAM (5'-3' sequence)	CGCCAGGAAAGTAGAAGGTCA
Reverse primer (5'-3' sequence)	AAAAGAAAACATAGATGGCAAAAGA

Table 2: Primers used for fragment analysis

Based on in-silico PCR (https://genome.ucsc.edu/cgi-bin/hgPcr), the resulting PCR product is:
>chr15:45361160+45361390 231bp

CGCCAGGAAAGTAGAAGGTCAAAAAGAAAACATAGATGGCAAAAGACGCCAGGAAAGTAGAAGGTCAgctggg
gtgcaagccggaagtcaccatcgagtgcacgggggcagaggcctccatccaggcgggcatctacgtgagtgggctgagggcagctttggggaatc
agcatagggagtgaaggaggcagaagtagggagtcaaacttctttaccagcttgctgcgtgagccccaagccaaacttattcaTCTTTTGCCTC
TATGTTTTCTTTT

Fragment analysis was aimed at the detection of the c.757del. Therefore, the wild type allele should be 231bp in length, and the mutant allele should be one bp shorter – 230bp.

Standard PCR thermal cycling parameters were used, with annealing temperature 60°C and 30 cycles. For PCR mix chemistry from our local distributor was used – CombiPPP (http://www.top-bio.cz/pcr-master-mixy-12.html?combi-ppp-master-mix). Size standard GS1200 LIZ (https://www.thermofisher.com/order/catalog/product/4379950#/4379950) was used. Products were separated on an ABI 3130 capillary sequencer (https://www.thermofisher.com/order/catalog/product/313001R#/313001R) and analysed with GeneMapper software v4.0 (https://www.thermofisher.com/order/catalog/product/4440915#/4440915).

Results are shown in Figure 2.

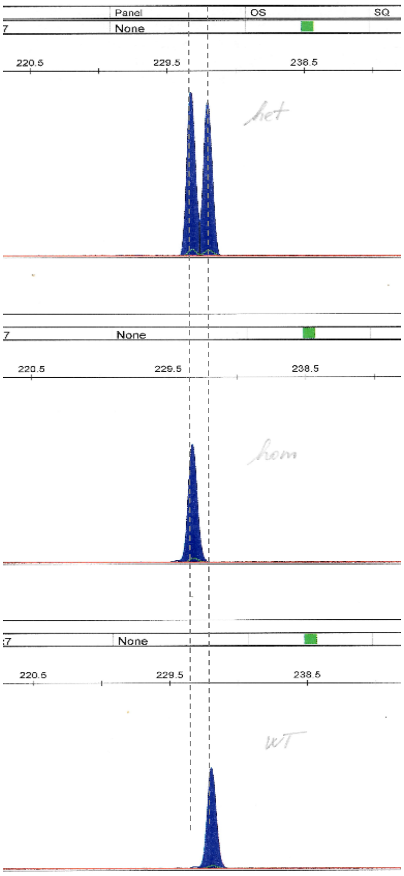

Figure 2: Fragment analysis results

Legend: Three samples are shown. The sample in the upper part represents a heterozygous carrier of c.757del on one allele and a wild type on the second allele of the SORD gene. The sample in the middle part is homozygous for the c. 757del. The sample in the lower part is a wild type control.

Our forward primer took advantage from the SNP: Chr15(GRCh38):g.45068982A>T , which was supposed to be A in gene and T in the pseudogene sequence. The SNP was located at the 3' end of the primer for the highest possible specificity. This SNP has a stated frequency in gnomAD genomes: 25%, but in the original paper it was used for gene/pseudogene discrimination [1]. The gnomAD frequency could have been explained by misalignment due to high gene/pseudogene homology. However, later on we learned that this SNP is also present in the gene sequence with a relatively high frequency (17% in our samples). Therefore, we cannot recommend to use our fragment analysis protocol, as serious allelic dropout might result. For screening of the c.757del variant, Sanger sequencing of exon 7 with gene specific primers and with the exclusion of the SORD2P is the best option we can recommend.

1.2.2. Sanger sequencing of the *SORD* gene

These PCR primers were used in our study (Table 3):

Primer	5'-3' sequence
SORD_ex9_R	CCCTGAGATCCCAAGACTG
SORD_ex9_F	CACCTGGCTCTTTCCTCTTG
SORD_ex8_R_new	TCCTCCCATTCTTTAACTGGC
SORD_ex8_F	TCCCGCTCAGTTAAGTTTGG
SORD_ex7_R_SQ_new	GCTCACGCAGCAAGCTGGTAAAG
SORD_ex7_R	AGCCTGGGCGACTGAGTGAG
SORD_ex7_F	TGAGTCATCAGATTTCTCTTGTTTG
SORD_ex6_R	GCTGTTTCCCAGTCAAGGAG
SORD_ex6_F	ATGTTTAATATTTCACGAACATATTCC
SORD_ex5_R	GTTCCCTGAATTCCCAGTCA
SORD_ex5_F	CGTGGCCATGTTAACTCCTT
SORD_ex4_R	CGAGGTCATTGTTGTTATGACG
SORD_ex4_F	GCATGCAAGCCTTCATAACA
SORD_ex3_R	TCTTGTTCCCTGCTGTACCC
SORD_ex3_F	ACCTTTTCTCATAAATAGATACGAATCC
SORD_ex2_R	GCAGTAGACTCTGTTCTCAGCCTAAC
SORD_ex2_F	AGCGTGCCATTTAGCGTATC
SORD_ex1_R	AGTGAGGCAGGATCGGTATG
SORD_ex1_F	CAGGCTGGCACAAAGGAG

Table 3: Primers used in this study

For primers from the original publication [1] for ex8 (5'-3' sequence: Forward - TCCCGCTCAGTTAAGTTTGG and reverse- GCTTCAAAATCCCCTCCTTC) two products are amplified:

>chr15:45072130+45072670 541bp length
>chr15_KI270849v1_alt:152235-153076 842bp length

With newly designed reverse primer (5'-3' sequence: Forward - TCCCGCTCAGTTAAGTTTGG and reverse- TCCTCCCATTCTTTAACTGGC), one PCR product for ex8 is amplified:

>chr15:45072130+45072627 498bp
This is illustrated below (from https://genome.ucsc.edu/cgi-bin/hgPcr):

A.

>chr15:45072130+45072670 541bp TCCCGCTCAGTTAAGTTTGG GCTTCAAAATCCCCTCCTTC
TCCCGCTCAGTTAAGTTTGGttggcaggttgagcagttcagcagtgtaca caaatcacattctcaggaatttttggatgctcagctgatgcaaccagaag
atacttgtgtgatgacaatatgttattgtaagtgggaagtcagtatttct tatcttctgagctttcttgtttttacctcctttacaggccactcgctctg
gtgggaacctcgtgcttgtggggctgggctctgagatgaccaccgtaccc ctactgcatgcagccatccgggaggtggatatcaagggcgtgtttcgata
ctgcaacacgtgagtatgccgtgggtgagccgggatgcccagcctccagc aagaccatggcaggccccactcagcctctgggcccatgagtctctgcctg
tttgttcatggggggcactccctggccacactgatagctgtgtgatataa cagggatccaaagagagaacactcactgccagttaaagaatgggaggaac
agaaggatggatggagggaggGAAGGAGGGGATTTTGAAGC

>chr15_KI270849v1_alt:152235-153076 842bp TCCCGCTCAGTTAAGTTTGG GCTTCAAAATCCCCTCCTTC
TCCCGCTCAGTTAAGTTTGGttggcaggttgagcagttcagcagtgtaca caaatcacattctcaggaatttttggatgctcagctgatgcaaccagaag
atacttgtgtgatgacaatatgttattgtaagtgggaagtcagtatttct tatcttctgagctttcttgtttttacctcctttacaggccactcgctctg
gtgggaacctcgtgcttgtggggctgggctctgagatgaccaccgtaccc ctactgcatgcagccatccgggaggtggatatcaagggcgtgtttcgata
ctgcaacacgtgagtatgctgtgggtgagccgggatgcccagcctccagc aagaccatggcaggccccactcagcctctggcccatgagtctctgcctgt
ttgttcatggggggcactccctggccacactgatagctgtgtgatataac agggatccaaagagagaacactcactcccaattaaagaatgggaaggccg
ggcgtggcggctcatgcctgtaatcccagcactttgggaggccgaggcag gcggatcacgaggtcaggagatcgagagcatcctggctaacacggtgacc
cctgtctctactaaaaatacaaaaaattagccaggcatggtggtgggtgt ctgtagtcccagctacttgggatgctgaggcaggagagtggtgtgaaccc
aggaggcagagattgcagtgagcggagatcgcaccactgcactccagcct gggcaacagagccagactctgtctcaaaaaaaaaaaaaagaatgggaggaa
cagaaggatggatggagggaggGAAGGAGGGGATTTTGAAGC

B.

UCSC In-Silico PCR

>chr15:45072130+45072627 498bp TCCCGCTCAGTTAAGTTTGG TCCTCCCATTCTTTAACTGGC
TCCCGCTCAGTTAAGTTTGGttggcaggttgagcagttcagcagtgtaca
caaatcacattctcaggaatttttggatgctcagctgatgcaaccagaag
atacttgtgtgatgacaatatgttattgtaagtgggaagtcagtatttct
tatcttctgagctttcttgtttttacctcctttacaggccactcgctctg
gtgggaacctcgtgcttgtggggctgggctctgagatgaccaccgtaccc
ctactgcatgcagccatccgggaggtggatatcaagggcgtgtttcgata
ctgcaacacgtgagtatgccgtgggtgagccgggatgcccagcctccagc
aagaccatggcaggccccactcagcctctgggcccatgagtctctgcctg
tttgttcatggggggcactccctggccacactgatagctgtgtgatataa
cagggatccaaagagagaacactcactGCCAGTTAAAGAATGGGAGGA

Primer Melting Temperatures

Forward: 60.2 C tcccgctcagttaagtttgg
Reverse: 60.4 C tcctcccattctttaactggc

*Figure 3**: PCR products for exon 8 of the SORD/SORD2P***

Legend:

***PART A**.: PCR products gained with amplification with priemrs for exon 8 from the original publication by Cortese, et al. both, gene (SORD) and pseudogene (SORD2P) exon 8 is amplified.*

***PART B**.: Only one specific product for gene specific exon 8 is gained with our proposed primers.*

Figure 4 presents electrophoreograms for exon 7 of the SORD gene. And sequences are compared to the SORD2P (pseudogene) sequence. Four differences used for discrimination are illustrated (red rectangles).

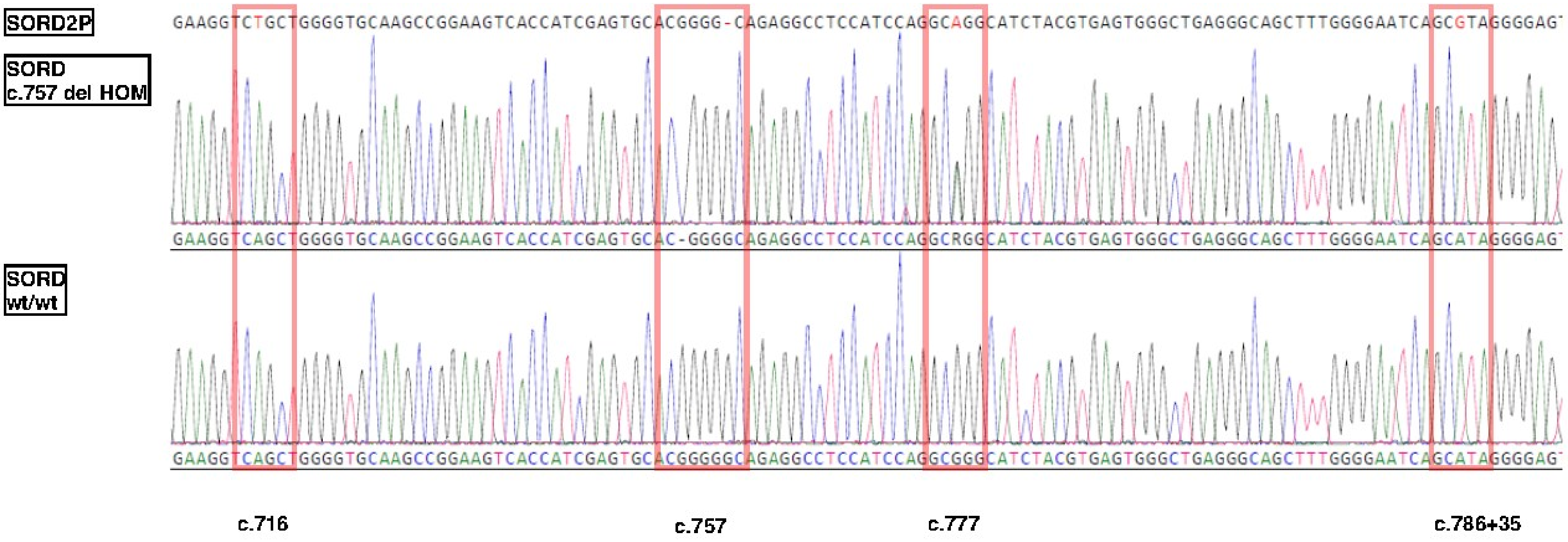

*Figure 4: **Electropherograms of SORD exon 7***

Legend:

Exon 7 of the SORD gene, a homozygote for the c.757del is shown in the upper part , the wild-type sequence is shown in the lower part of the figure. Pseudogene sequence is stated in letters above the electropherograms. Differences are highlighted in with red rectangles.

1.2.3. Analysis of WES data

For samples that went through WES analysis, we present results from analysis, to discuss which different variants in the known neuropathy genes were seen. Criteria for filtering are in the first line of the table. As data in the table presents, no other interesting variants, that could possibly explain the cause of the disease, were detected with WES in those three families. The evaluation and conclusion for each of the variants is in the first row of the table.

Filters:	Function: exonic or splicing; GnomAD Exome all: frequency less than 0.01%; OMIM related to Inherited peripheral neuropathy										
							**Fam 5**				
	Chr	Start	End	Ref	Alt	gNomen	Func.refGe	Gene.refG	ExonicFunc.refGene	AAChange.refGene	Genotype
SORD	chr15	45357501	45357501	C	A	chr15:45357501C>A	exonic	SORD	nonsynonymous SNV	SORD:NM_003104:exon5:c.C458A:p.A153D	HET
SORD	chr15	45361217	45361217	G	-	chr15:45361217G>-	exonic	SORD	frameshift deletion	SORD:NM_003104:exon7:c.753delG:p.A253Qfs*27	HET
UTR5	chr12	57979190	57979190	C	G	chr12:57979190C>G	UTR3	KIF5A	NM_004984:c.*1226C>G;NM_001354705:c.*1226C>G		HET
							**Fam 13**				
	Chr	Start	End	Ref	Alt	gNomen	Func.refGe	Gene.refG	ExonicFunc.refGene	AAChange.refGene	
different phenotype (HSN), autosomal recessive phenotype, only one variant in heterozygous state, second variant is missing	chr6	56434712	56434712	G	T	chr6:56434712G>T	exonic	DST	nonsynonymous SNV	DST:NM_015548:exon35:c.C5951A:p.P1984H,DST:NM_183380:exon45:c.C6929A:p.P2310H,DST:NM_001144770:exon46:c.C7049A:p.P2350H,DST:NM_001144769:exon48:c.C7463A:p.P2488H	HET
different phenotype (HSN), autosomal dominant, ClinVar: likely bening, GnomAd frequency: 0.21%	chr12	994324	994324	A	G	chr12:994324A>G	exonic	WNK1	nonsynonymous SNV	WNK1:NM_014823:exon17:c.A3613G:p.T1205A,WNK1:NM_001184985:exon19:c.A5134G:p.T1712A,WNK1:NM_018979:exon19:c.A4354G:p.T1452A,WNK1:NM_213655:exon19:c.A5110G:p.T1704A	HET
SORD	chr15	45361217	45361217	G	-	chr15:45361217G>-	exonic	SORD	frameshift deletion	SORD:NM_003104:exon7:c.753delG:p.A253Qfs*27	HOM
This is a known rare polymorphism in the PMP22 - not causal (population frequency is 0.42%, we have seen this variant repeatedly as not causal for CMT phenotype	chr17	15134364	15134364	G	A	chr17:15134364G>A	exonic	PMP22	nonsynonymous SNV	PMP22:NM_153322:exon4:c.C353T:p.T118M,PMP22:NM_000304:exon5:c.C353T:p.T118M,PMP22:NM_001281455:exon5:c.C353T:p.T118M,PMP22:NM_001281456:exon5:c.C353T:p.T118M,PMP22:NM_153321:exon5:c.C353T:p.T118M	HET
heterozygous for one missence variant, variant on the second allele is not present, PRX are mostly frameshift (non-sense) variants	chr19	40903436	40903436	G	T	chr19:40903436G>T	exonic	PRX	nonsynonymous SNV	PRX:NM_181882:exon7:c.C823A:p.L275I	HET
different phenotype : IPN + deafness, autosomal recessive inheritance, only one heterozygous variatnt, no second variant	chr19	50766582	50766582	C	T	chr19:50766582C>T	exonic	MYH14	nonsynonymous SNV	MYH14:NM_024729:exon20:c.C2476T:p.R826C,MYH14:NM_001077186:exon21:c.C2500T:p.R834C,MYH14:NM_001145809:exon22:c.C2599T:p.R867C	HET
							**Fam 10**				
	Chr	Start	End	Ref	Alt	gNomen	Func.refGe	Gene.refG	ExonicFunc.refGene	AAChange.refGene	
noncoding exon	chr10	64578298	64578298	-	A	chr10:64578298->A	splicing	EGR2			HET
NM_015915.4(ATL1):c.723+3_723+19del, intronic deletion	chr14	51080068	51080084	AGGTT	-	chr14:51080068AGGTTTG	exonic	ATL1	frameshift deletion	ATL1:NM_015915:exon7:c.722_723del:p.K241Sfs*7,ATL1:NM_181598:exon7:c.722_723del:p.K241Sfs*7,ATL1:NM_001127713:exon8:c.722_723del:p.K241Sfs*7	HET
SORD	chr15	45357501	45357501	C	A	chr15:45357501C>A	exonic	SORD	nonsynonymous SNV	SORD:NM_003104:exon5:c.C458A:p.A153D	HET
SORD	chr15	45361217	45361217	G	-	chr15:45361217G>-	exonic	SORD	frameshift deletion	SORD:NM_003104:exon7:c.753delG:p.A253Qfs*27	HET

Table 4: Other variants detected with WES in patients with biallelic SORD disease causing variants.

References:

1. Cortese, A., et al., *Biallelic mutations in SORD cause a common and potentially treatable hereditary neuropathy with implications for diabetes.* Nat Genet, 2020. **52**(5): p. 473-481.
